# Perceptions reported by residents in psychiatry on oral health problems in their patients with severe mental disorders: a qualitative study at a Brazilian university specialized psychiatric service

**DOI:** 10.1192/j.eurpsy.2022.2197

**Published:** 2022-09-01

**Authors:** L. Guerra, E. Turato, R. Bastos, B. Gondinho, F. Silva, J. Cavalcante

**Affiliations:** State University of Campinas, Laboratory Of Clinical-qualitative Research, Campinas, Brazil

**Keywords:** training in psychiatry, severe mental disorder, oral health, Qualitative research

## Abstract

**Introduction:**

There is a greater prevalence of oral problems in patients suffering from severe mental illness than in the general population. The psychiatrist use to be, naturally, a health professional with great clinical influence over these patients. Do young psychiatrists in training include oral evaluations on their patients? How does this doctor perceive oral health care in the context of follow-up of people with chronic mental disorders?

**Objectives:**

To interpret the meanings of the practice or not, regarding oral health guidelines, as reported by residents in psychiatry working in care and follow-up services to patients with severe disorders at a public university.

**Methods:**

Clinical-qualitative design. Semi-directed interviews with open-ended questions in-depth carried out with six participants. Sample closed by saturation information criterion. Residents see their patients at the General Hospital of the State University of Campinas. Interview material, audio-recorded and transcribed in full, was treated by Clinical-Qualitative Content Analysis, using concepts of theoretical framework from Medical Psychology. Interviewer was a female professor of dentistry.

**Results:**

From the discussion, two categories of analysis were selected for this presentation. (1) medical practice obeys the natural logic of construction of paradigmatic areas: historically, dentistry has created a care model with independence from medicine; (2) dentist is not called to participate in “collusion of anonymity”. This is an expression construct by the psychoanalyst Balint to describe the taking of relevant clinical decisions, without no professional assume the responsibility for these.

**Conclusions:**

These meanings may guide changes in professional conduct as well as in the curriculum of medical training programs.

**Disclosure:**

No significant relationships.

